# Proteomics reveals disturbances in the immune response and energy metabolism of monocytes from patients with septic shock

**DOI:** 10.1038/s41598-021-94474-0

**Published:** 2021-07-26

**Authors:** Pedro Mendes de Azambuja Rodrigues, Richard Hemmi Valente, Giselle Villa Flor Brunoro, Helder Takashi Imoto Nakaya, Mariana Araújo-Pereira, Patricia Torres Bozza, Fernando Augusto Bozza, Monique Ramos de Oliveira Trugilho

**Affiliations:** 1 National Institute of Infectious Diseases Evandro Chagas, Fiocruz, Rio de Janeiro, 21040-360 Brazil; 2 Laboratory of Toxinology, Oswaldo Cruz Institute, Fiocruz, Rio de Janeiro, 21040-900 Brazil; 3 School of Pharmaceutical Sciences, University of São Paulo, São Paulo, 05508-000 Brazil; 4 Laboratory of Immunopharmacology, Oswaldo Cruz Institute, Fiocruz, Rio de Janeiro, 21.040-900 Brazil; 5 Center for Technological Development in Health, Fiocruz, Rio de Janeiro, 21040-361 Brazil

**Keywords:** Immunology, Pathogenesis, Immunopathogenesis, Infection, Inflammation

## Abstract

Sepsis results from a dyshomeostatic response to infection, which may lead to hyper or hypoimmune states. Monocytes are central regulators of the inflammatory response, but our understanding of their role in the genesis and resolution of sepsis is still limited. Here, we report a comprehensive exploration of monocyte molecular responses in a cohort of patients with septic shock via proteomic profiling. The acute stage of septic shock was associated with an impaired inflammatory phenotype, indicated by the down-regulation of MHC class II molecules and proinflammatory cytokine pathways. Simultaneously, there was an up-regulation of glycolysis enzymes and a decrease in proteins related to the citric acid cycle and oxidative phosphorylation. On the other hand, the restoration of immunocompetence was the hallmark of recovering patients, in which an upregulation of interferon signaling pathways was a notable feature. Our results provide insights into the immunopathology of sepsis and propose that, pending future studies, immunometabolism pathway components could serve as therapeutic targets in septic patients.

## Introduction

Sepsis represents a significant burden for healthcare systems worldwide due to its high incidence, mortality, and associated costs^1^. The critical event for the development of sepsis seems to be a dysregulation of the host response to infection, resulting in a state of life-threatening loss of homeostasis. However, despite decades of research, a clear pathobiological framework of sepsis is still lacking, and no specific immunomodulatory treatment is available in the clinical setting^2^.

Our understanding of the course of the immune response in sepsis is scarce. Traditionally, it is described as biphasic: after the initial recognition of pathogens by the innate immune system, which may trigger a systemic hyperinflammatory state, unregulated inflammatory resolution mechanisms can induce subsequent immunosuppression, resulting in protracted or recurrent infections^3^. More recently, an alternative model postulates that sepsis results in a persistent inflammatory activation and suppression of adaptive immunity^4^. Knowledge of the link between this pathological immune response and organ dysfunctions is also limited and may involve direct inflammatory tissue damage and microcirculatory disturbances. Intriguingly, although in shock states the main cause of organ failure is a reduction in oxygen delivery, in septic shock, oxygen consumption may remain impaired despite restored tissue perfusion. This observation led to the hypothesis that sepsis-induced primary bioenergetic alterations may be relevant disease mechanisms^5,6^.

Monocytes are key coordinators of inflammation. When activated, they can rapidly migrate to tissues and differentiate into macrophages and dendritic cells. Also, monocytes act as direct effectors of innate immunity, exhibiting phagocytic microbicidal activity, producing inflammatory mediators, presenting antigens, and influencing the adaptive immune response^7,8^. Therefore, these cells are potential critical elements for the genesis and resolution of sepsis. Experimentally, it is possible to induce a polarization of mononuclear phagocytes into pro-inflammatory (M1) or anti-inflammatory (M2) subsets. Curiously, the M1 phenotype is dependent on a metabolic shift from oxidative phosphorylation to glycolysis, termed the "Warburg effect" ^9,10^. Interestingly, all effector T cell subsets undergo similar metabolic alterations upon activation^11,12^. Although the M1/M2 paradigm has been used as a surrogate for disease states, mononuclear phagocytes exhibit a high degree of immune phenotypical plasticity in response to complex environmental stimuli^13,14^, which may also correspond to particular metabolic alterations^15^. Hence, it is important that disease-specific phenotypes be characterized directly from clinical samples.

Currently, omics technologies have a consolidated role among the tools employed in sepsis translational research. To this date, most omics-based studies carried-out in the clinical setting aimed at the characterization of syndrome-related biomarkers^16^. As such, they primarily sought molecules that have a reproducible association with diagnosis or clinical outcomes, and inferences about the disease biology are often limited or absent. To this last end, proteomics has inherent advantages over transcriptomics, as proteins are the main effectors of most cellular processes, and post-transcriptional regulation often renders RNA and corresponding protein levels uncorrelated^17^.

In this study, we explore the proteome of monocytes in sepsis through a discovery-driven approach. Using samples from patients in the acute and recovery phases, we applied functional analysis methods to provide fresh insights into disturbed cellular mechanisms, which revealed that alterations in energy metabolism and inflammatory pathways are prominent molecular features in monocytes of patients with septic shock.

## Results

### Study population

Nine patients with septic shock and six healthy volunteers (Control group) were included, whose demographic and clinical characteristics are shown in Supplementary Table S1. Age and sex were similar between groups. Blood samples were collected from patients in the acute phase (Sepsis group), within 72 h of hospitalization, and in the recovery phase (Recovery group), before discharge from the intensive care unit (ICU). The infection sites were equally distributed between pulmonary, abdominal, and urinary tract. All patients in the Sepsis group had hemodynamic shock, two-thirds required mechanical ventilation, and one-third required renal replacement therapy. The ICU stay was, on average, 19 days. Despite an average SOFA of 11 for the Sepsis group, there were no deaths in 28 days and only one late hospital death.

### Measurement of monocyte proteomes

**Figure 1:**
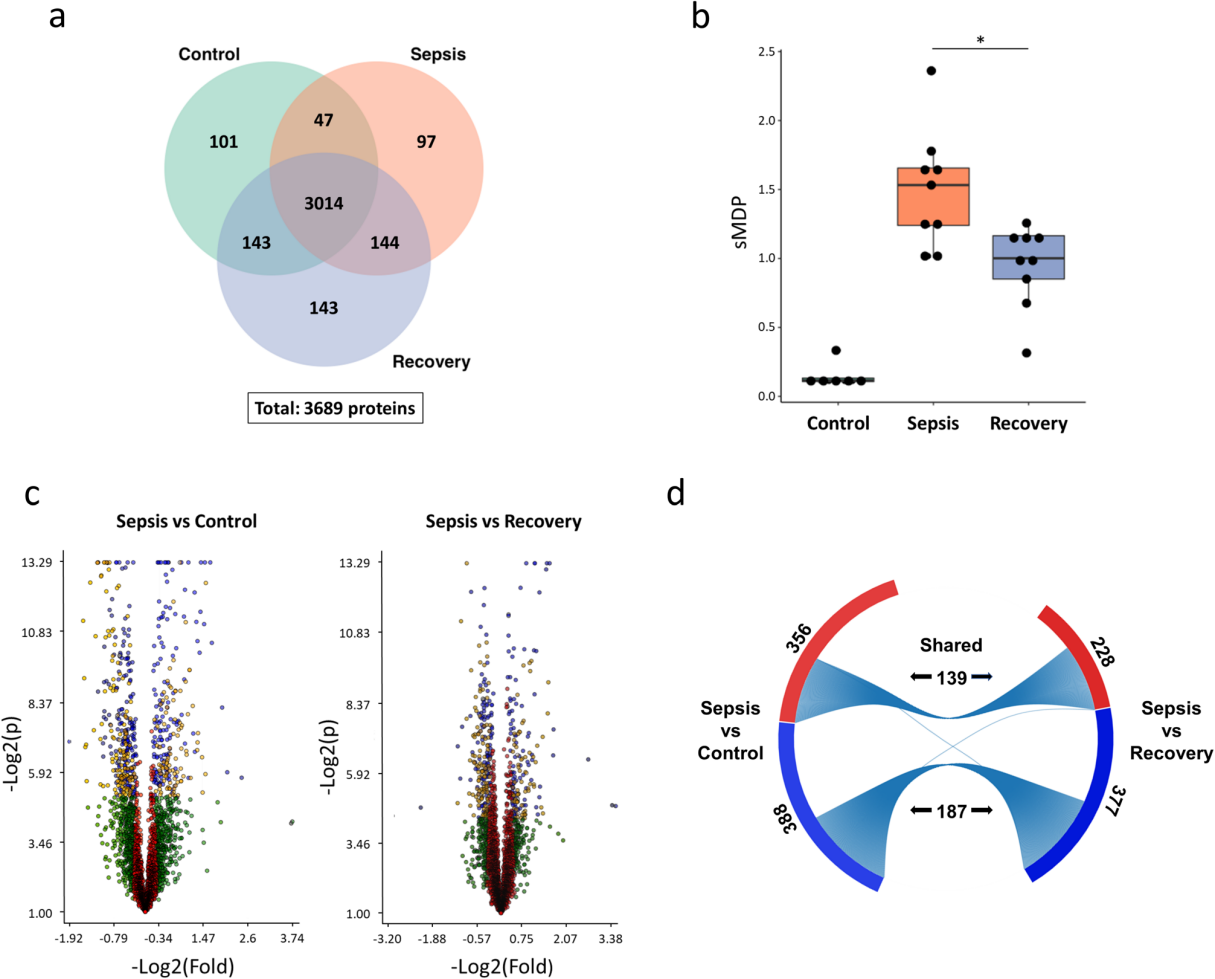
Alterations in the proteome of monocytes in the acute phase of sepsis (Sepsis group), compared with the recovery phase (Recovery group) and healthy subjects (Control group). (**a**) Total, unique, and shared protein identifications among the experimental groups. (**b**) Molecular degree of perturbation of the Sepsis end Recovery groups in relation to the Control group. The plot represents individual values with median and IQR per group. (**c**) Volcano plot of all shared proteins in the comparisons between the Sepsis and Control and Sepsis and Recovery groups. Each dot represents a protein mapped according to its log2 (fold change) on the y-axis and its − log2 (t-test *p* value) on the x-axis. The red dots indicate proteins that satisfy neither the fold-change cutoff nor the *p* value (0.05). Green dots depict protein entries that meet the fold-change cutoff but not the *p* value. Orange dots indicate proteins that satisfy both fold-change and *p* value but have low abundances, as determined by an additional stringency filter. Blue dots represent protein entries that met all statistical filters and were selected for further analysis. (**d**) Total and shared proteins significantly up (red bars) or down (blue bars)-regulated in the comparisons between the Sepsis and Control and Sepsis and Recovery groups.

A shotgun proteomics (LC–MS/MS) approach was applied to obtain comprehensive coverage of monocytes' proteins isolated from the blood. We were able to confidently identify (FDR < 1%) 40,447, 43,016, and 48,339 peptides, with inference by maximum parsimony of 3454, 3496, and 3716 proteins in the control, sepsis, and recovery groups, respectively (Supplementary Table S2 and Supplementary File 1). A total of 3689 proteins were identified—considering only those present in multiple biological replicates—with 3014 proteins in common among all groups and 101, 97, and 143 proteins characterized exclusively in Control, Sepsis, and Recovery groups, respectively (Fig. 1a).

The molecular degree of perturbation (MDP) score, which measures the proteome's alteration compared to healthy controls, demonstrated a clear separation between individuals from both Sepsis and Recovery groups and the Control group (Fig. 1b). Although there is some overlap between individuals of the Sepsis and Recovery groups, the sepsis group's median MDP was significantly higher, indicating that sepsis induces significant monocyte proteome changes. It also highlights the differences between the Control and Recovery groups: the first was composed of healthy volunteers and the former of patients who overcame the acute phase of organ dysfunctions but were still under hospital treatment.

To study the alterations that reflect sepsis progression, we worked with comparisons between the Sepsis and Control and Sepsis and Recovery groups (isoforms were discounted): the number of proteins showing statistically significant differences was 268 and 182, respectively (Fig. 1c and Supplementary File 2). For the functional analysis of the proteomes, we also included proteins detected exclusively in one group. Although there is no *p* value assigned to each protein abundance comparison, we argue that since multiple biological and technical replicates were analyzed and such proteins were only detected in one biological condition, this strongly suggest differential regulation abundance. These comprised 485 and 430 proteins in the comparisons between the Sepsis and Control and Sepsis and Recovery groups, respectively. Each comparison revealed a unique expression profile, as most of the up and downregulated proteins were not coincident (Fig. 1d).

### Functional analysis of differentially regulated proteins

**Figure 2:**
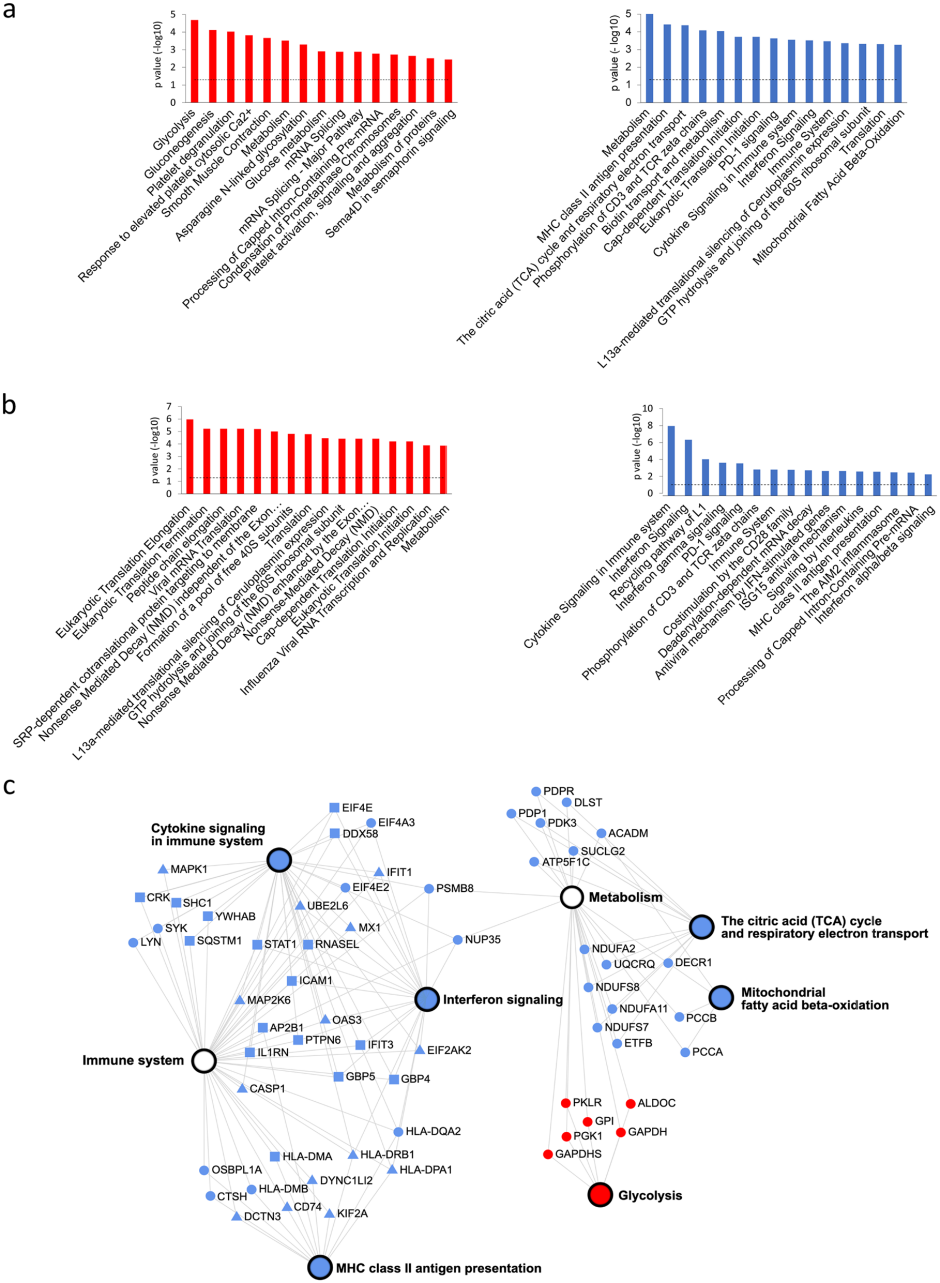
Functional analysis of the sepsis monocyte proteome. Enriched Reactome pathways among the differentially expressed proteins in groups (**a**) Sepsis, compared to Control and; (**b**) Sepsis, compared to Recovery. The y-axis represents the − log10 of the *p* value for the association between the pathways and the set of proteins up (red bars) or down (blue bars)-regulated. Significance (dotted lines) was defined as *p* ≥ 0.05 (Benjamini and Hochberg); (**c**) A network of pathways selected from (**a**,**b**). Nodes represent proteins up (red) or down (blue)-regulated in the comparisons between Sepsis and Control (circles), Sepsis end Recovery (squares), or both comparisons (triangles).

We used the InnateDB platform^18^, with the annotations imported from the Reactome database^19^, to explore the biochemical pathways statistically over-represented on the differentially regulated abundant proteins of the experimental groups (Fig. 2a–c). Among the up-regulated proteins in the Sepsis group compared to the Control group, the pathways with the highest statistical significance were related to glycolytic metabolism, including the canonical enzymes PGK1, ALDOA, ALDOC, GADPH, PKLR GPI, as well as LDHA, responsible for converting pyruvate into lactate in situations where oxidative phosphorylation decreases. On the other hand, proteins down-regulated in sepsis were associated with oxidative phosphorylation and Krebs cycle pathways (ATP5C1, DLST, ETFB, NDUFA11 NDUFA2, NDUFS7, NDUFS8, PDK3, PDP1, PDPR, RXRA, SUCLG2, TACO1 and UQCRQ), beta-oxidation of fatty acids (ACADM, DECR1, PCCA, PCCB), and, in parallel, to the MHC class II antigen presentation pathway (CD74, CTSH, DCTN3 DYNC1LI2, HLA-DMA, HLA-DMB, HLA- DPA1, HLA-DQA2, HLA-DRA, HLA-DRB1, KIF2A, OSBPL1A) and the related interferon signaling pathway (EIF2AK2, EIF4A3, EIF4E2, HLA-DPA1, HLA-DQA2, HLA-DRA, HLA-DRB1, IFIT1, MX1, NUP35, OAS3, PSMB8, UBE2L6). This immune profile was compatible with the results of flow cytometry experiments on the surface expression of HLA-DR in patients from the participant ICUs ( Supplementary Figure S1). The analysis of positively regulated proteins in the Recovery group in relation to the Sepsis group revealed a high representation of proteins associated with cytokine signaling (CASP1, CRK, DDX58, EIF2AK2, EIF4E, GBP4, GBP5, HLA-DPA1, HLA-DRA, HLA- DRB1, ICAM1, IFIT1, IFIT3, IL1RN, MAP2K6, MAPK1, MX1, OAS3, PTPN6, RNASEL, SHC1, SQSTM1, STAT1, UBE2L6, YWHAB), highlighting the interferon pathway (GBP4, GBP5, HLA-DPA1, HLA-DPA1, HLA-DPA1, HLA-DPA1, HLA-DPA1, HLA-DPA1, HLA—DRA HLA-DRB1, ICAM1, OAS3, PTPN6) and the presentation of MHC class II antigens (AP2B1 CD74, DCTN3, DYNC1LI2, HLA-DMA, HLA-DPA1, HLA-DRA, HLA-DRB1, KIF2A).

### Immunometabolic profile of the experimental groups

**Figure 3:**
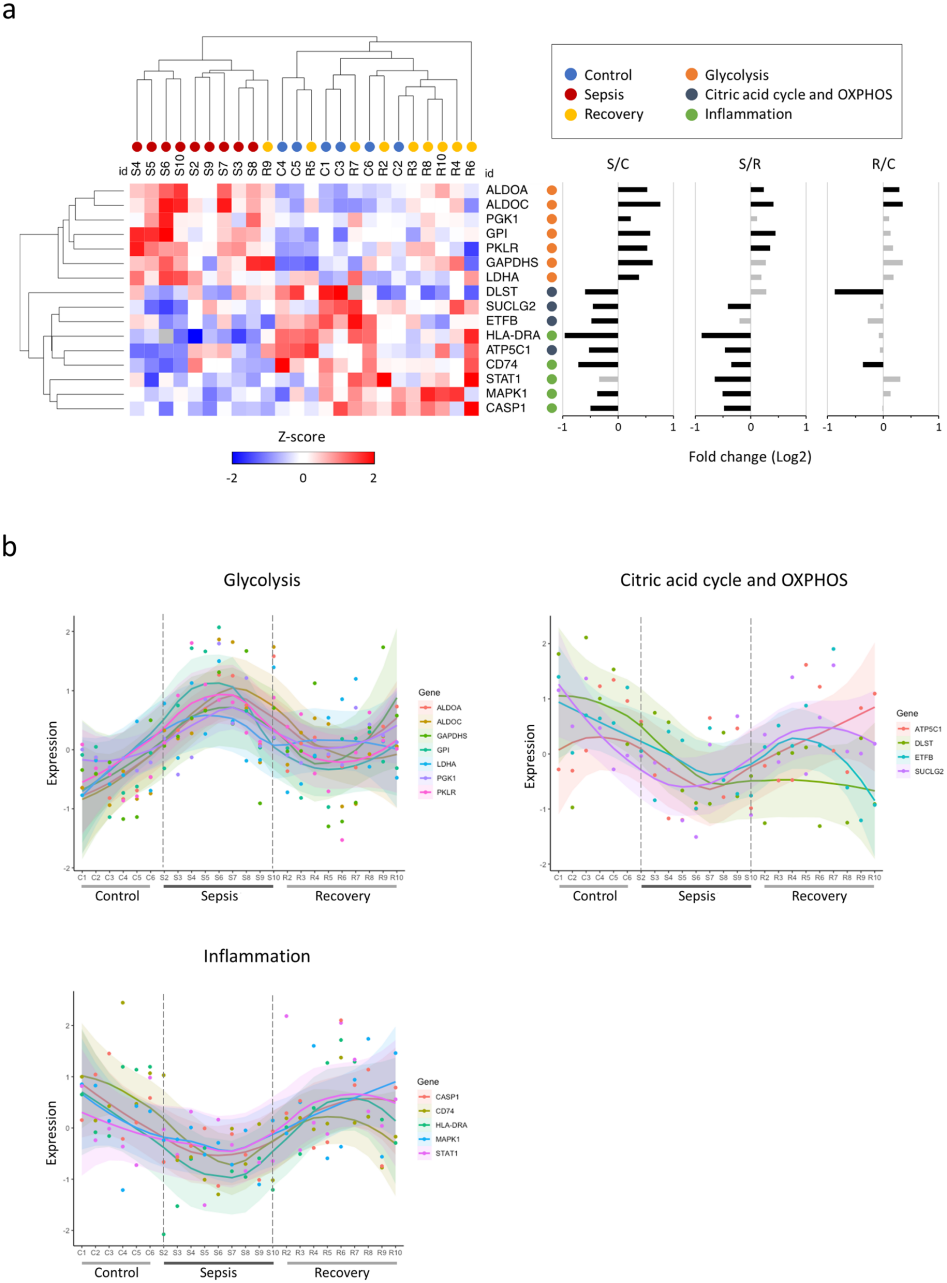
Immunometabolic profile of the experimental groups. (**a**) On the left, the columns (individuals) and rows (proteins) of the heatmap were clustered hierarchically by the Euclidean distance. The expression values were normalized by the z-score of each line. On the right, the fold changes of the protein abundance ratios (NSAF) between the control (C), Sepsis (S), and Recovery (R) groups are displayed. The gray bars represent statistically non-significant differences (TFold); (**b**) Expression (z-scores) of proteins separated by function. The samples were organized on the y axis by the identifier of individuals in the Control group (C1 to C6) followed by the Sepsis (S2 to S10) and Recovery (R2 to R10) groups. Each dot represents a protein with the corresponding smooth line (loess regression) showing each group's expression trends.

Since annotations related to energy metabolism and the immune response featured prominently on the enrichment analysis, we selected the proteins most specifically associated with those pathways and with high coverage (> 90%) on the experimental groups for a detailed expression profiling (Fig. 3a,b). Among glycolysis proteins, glucose-6-phosphate isomerase (GPI), aldolase (ALDOA, ALDOC), glyceraldehyde-3-phosphate dehydrogenase (GAPDHS), phosphoglycerate kinase (PGK1), and pyruvate kinase (PKLR) are involved in the main chain of reactions of the conversion of glucose to pyruvate (steps 2, 4, 6 and 7, and 10 respectively). Lactate dehydrogenase (LDHA) catalyzes the interchange between pyruvate and lactate. Of the citric acid cycle, dihydrolipoyl succinyltransferase (DLST) is a component of the α-ketoglutarate-dehydrogenase complex that oxidizes α-ketoglutarate to succinyl-CoA and CO2. In sequence, the conversion of succinyl-CoA to succinate is catalyzed by succinyl-CoA ligase (SUCLG2). From oxidative phosphorylation, electron-carrying flavoprotein (ETFB) transfers electrons from mitochondrial dehydrogenases to the electron transport chain, which produces an electrochemical gradient used by ATP mitochondrial synthase (ATP5C1) to catalyze ATP synthesis. Of the proteins involved in the inflammatory response, the signal transcription activating factor and activation transducer-1 (STAT1) is a mediator of interferons' intracellular signaling, and the mitogen-1 activated protein kinase (MAPK1) participates in the regulation of transcription activated by growth factors and cytokines. The human leukocyte antigen-DR (HLA-DRA) is an MHC class II antigen-presenting cell surface molecule. The MHC class II (CD74) invariant chain participates in the stabilization and cell transport of these molecules.

Even though heterogeneity of protein regulation among biological replicates was evident—as expected for clinical samples—hierarchical clustering was able to completely separate individuals from the Control and Sepsis Groups. The Recovery group, except for one individual, was grouped with the Control group. Similarly, the proteins of the immune response, citric acid cycle, and oxidative phosphorylation were grouped separately from those related to glycolysis (Fig. 3a). In the Sepsis group, compared to the Control group, the glycolysis proteins showed a consistent positive regulation. In contrast, those of the inflammation, citric acid cycle, and oxidative phosphorylation were negatively regulated. These differences were largely reversed in the recovery group (Fig. 3a,b). Most of the differences in expression between the recovery and control groups were not statistically significant.

## Discussion

Here, we report the alterations in the proteomic phenotype of blood monocytes from patients with septic shock. Our investigation highlighted distinctive molecular profiles associated with energy metabolism and the immune response. In the acute stage of septic shock, the observed protein expression levels were indicative of a shift from oxidative phosphorylation to glycolysis, compatible with the Warburg effect. In parallel, these monocytes displayed characteristic features of an impaired inflammatory response, which reversed as patients recovered.

So far, most proteomics studies in clinical sepsis utilized plasma samples, and to our knowledge, none investigated monocytes^20^. In our experiments, isolated monocytes were analyzed through an MS-based workflow, which measured over 3600 proteins with high specificity across samples, providing a global assessment of cellular processes. Our experimental design included sampling at different time points, which allowed us to explore the dynamic nature of the immune response in sepsis. Furthermore, our methodology was unbiased by previously selected protein identities or known associations, which we argue is a valuable feature in studying diseases in which the molecular diagnosis and pathobiology are not yet clearly established. Our results indicate that septic shock induces comprehensive changes in the monocyte proteome, demonstrated by the molecular degree of perturbation of both the acute and recovery stages relatively to healthy individuals and the hundreds of differentially regulated abundant proteins between the experimental groups.

Immunometabolism, the interplay between cell metabolism and immunity, has already been shown to play a critical role in a wide range of clinical conditions such as autoimmune diseases, cancer, transplant and atherosclerosis^21,22^. In sepsis, the association between primary defects of cellular metabolism and organ dysfunctions is a well-recognized phenomenon. However, only a few studies sought to describe this link in the immune system. Early reports revealed an impaired mitochondrial function in peripheral blood mixed leucocyte populations in the clinical setting, due to inhibited ATP synthase activity or uncoupling of oxidative phosphorylation^23,24^. More recently, transcriptomics studies in larger cohorts reported overexpression of glycolytic genes in whole blood and total blood leukocyte samples^25–27^. In our study, a prominent finding of the pathway analysis in septic shock was an increase in monocyte proteins associated with glycolysis paired with a reduction in oxidative phosphorylation proteins. The positive regulation of most canonical glycolytic enzymes was joined by increased lactate dehydrogenase, implying a diversion in the glycolytic flow to produce lactate. Also, enzymes related to the beta-oxidation of fatty acids were negatively regulated, indicating a reduction in substrates' supply for the citric acid cycle. There was a consistent trend of reversal of this metabolic profile in the recovery stage, although not statistically significant for most proteins, which could reflect the patients' early convalescence status. These results suggest that a bioenergetic shift from oxidative phosphorylation to glycolysis in monocytes may be a characteristic of septic shock.

The dysregulated immune response in sepsis involves wide disturbances in both the innate and adaptive immune systems. The most validated biomarker of immune dysfunction in sepsis is the reduction of the expression of the MHC class II molecule HLA-DR on the surface of circulating monocytes, which is associated with an increase in the risk of infectious complications and death^28,29^. Our study observed a negative regulation of several MHC class II proteins, such as HLA-DR, HLA-DP, and HLA-DQ, in the acute phase of septic shock. Concordantly, there was a reduction of some of the central mediators of inflammatory activation, like MAPK, which participates in the TNF-α, IL-1, and TLR signaling, and Caspase-1, a point of convergence of the inflammasome activation, which generates the biologically active forms of IL-1 β and IL-18. This immune profile was reversed in the recovery phase, where there was a positive regulation of proteins related to cytokine signaling pathways, particularly IFN-ɣ, a potent proinflammatory stimulus. From these findings, we can conclude that, in our cohort, septic shock was associated with an impaired monocyte inflammatory profile. Although all subjects had clinical markers of severity, there were no sepsis-related deaths, which could be a result of the transition to a proinflammatory phenotype observed in the recovery stage.

It is noteworthy that our cohort's immunometabolic profile differs from those resulting from the in vitro M1 activation, where aerobic glycolysis accompanies a classic proinflammatory phenotype^9,10^. In this regard, aerobic glycolysis in the early stage of septic shock could be an event promoting the transition to immunocompetence observed in the recovery phase. Alternatively, it could represent a peculiar state resulting from the monocytes' adaptation to clinical sepsis, not mimicked by in vitro models. It would be interesting to assess whether this phenotype differs between patients with different clinical outcomes, which was not possible in our cohort since all patients survived sepsis and were discharged from the ICU.

As immunopathologic mechanisms unravel, ensuing therapies are proposed, which have so far included nonselective anti-inflammatory agents, administration or specific inhibition of inflammatory mediators, and the neutralization of microbial products^30^. In this regard, an interesting point for discussion from our results is the role of IFN-ɣ as a potential therapy in sepsis. Previous clinical studies demonstrated that IFN-ɣ treatment could restore monocytic HLA-DR expression, the secretion of TNF-α upon ex vivo LPS stimulation, and reduce infection-related mortality^31,32^. Hence, the upregulation of IFN-ɣ signaling observed in our recovering patients may have been essential to the restoration of the immune function and resolution of sepsis, highlighting the value of further investigation of this cytokine as a treatment in immunosuppressed sepsis populations.

Our study had several limitations. As is usual in studies based on clinical samples, our experimental design did not allow us to determine causal relationships between the disturbed cellular processes. However, the interdependence between aerobic glycolysis and immune activation states has already been well established in vitro in cells of the mononuclear phagocytic system, through, for example, pharmacological inhibition of glycolysis^33,34^. There were also limitations related, directly or indirectly, to technical issues. First, the relative abundance of proteins may not represent the functional activation of a pathway, especially regarding immune signaling, which depends on post-translational modifications such as phosphorylation. In this sense, phosphoproteomics or immunodetection techniques could represent an interesting, complement but would require another set of sample preparation and protein quantities greater than those obtained in the present study. Also, we included a relatively small number of patients, reflecting the time and resources required for our in-depth and cell-specific analytical goals. Given our experimental design's exploratory nature, it should be considered as a proof-of-concept study, which may serve as a foundation for further studies in larger cohorts using more focused protein-based methodologies.

**Figure 4:**
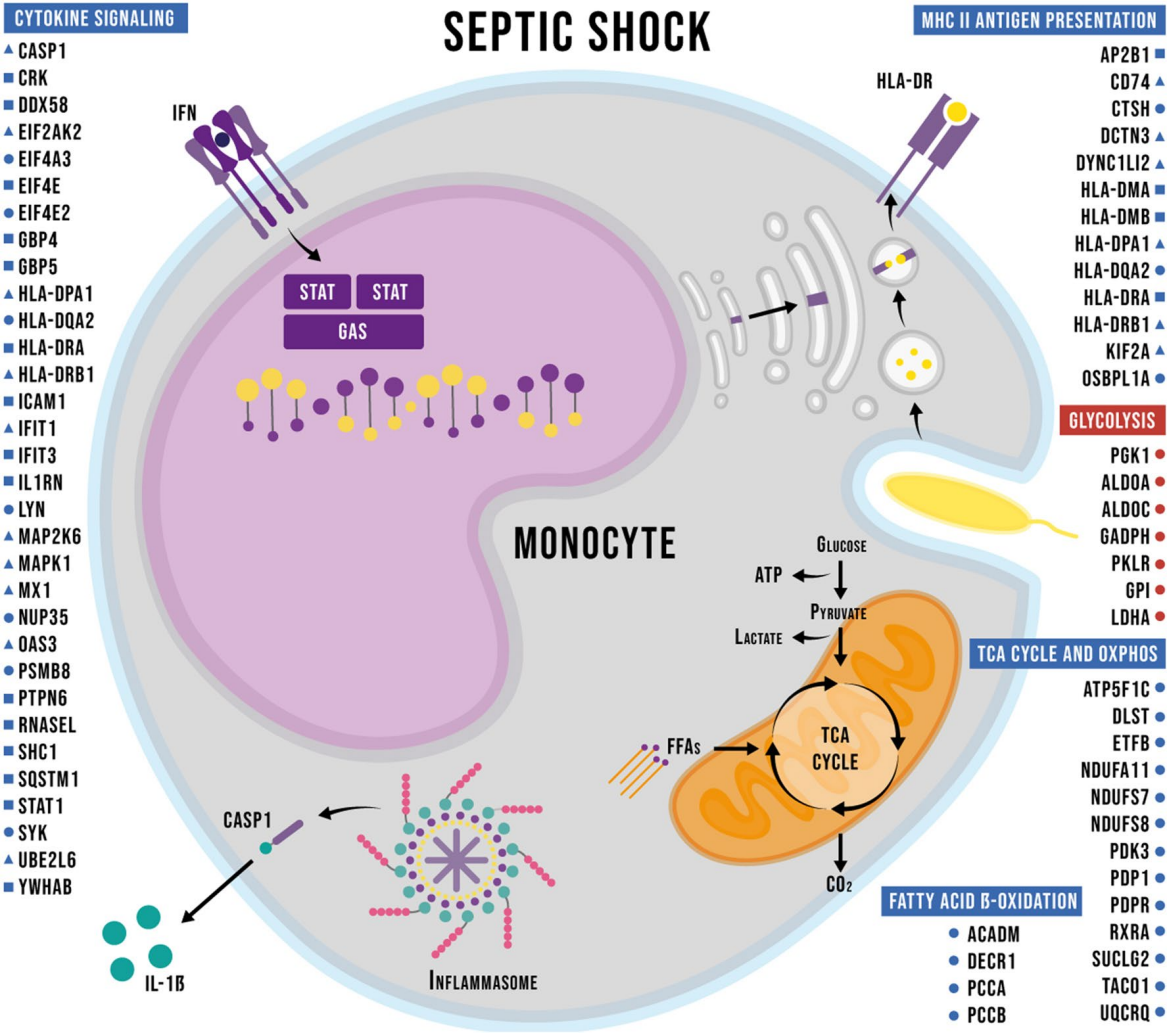
Graphical summary of the main findings regarding the immunometabolic phenotype. Proteins of selected enriched Reactome pathways are listed. Adjacent symbols indicate up (red) or down (blue)-regulation in the comparisons between Sepsis and Control (circles), Sepsis end Recovery (squares), or both comparisons (triangles).

In conclusion, this study indicates that the transition to aerobic glycolysis in monocytes may be an important clinical sepsis feature. Also, we were able to demonstrate the association of these metabolic changes with patients' immune activation states in the acute and recovery phases (Fig. 4). Our cohort profile was initially suggestive of immunosuppression, succeeded by the restoration of immunocompetence, which was documented at the molecular level by the positive regulation of proteins involved in the presentation of antigens and signaling by cytokines, and was accompanied by the clinical resolution of the infection and organic dysfunctions. Proteomics is a powerful yet underused tool for identifying molecular profiles that lead to biological phenomena characterization in clinical samples. We argue that obtaining large-scale data at the protein level is a valuable complement to the transcriptomics studies that dominate the field and may help us build more coherent and reliable pathophysiological models.

## Methods

### Study design and patients

We enrolled prospectively adult (≥ 18 years old) patients with sepsis from community-acquired infections, diagnosed according to the Third International Consensus Definitions for Sepsis and Septic Shock (Sepsis-3) criteria, admitted to the general intensive care units of the participant hospitals (Hospital São Lucas, Hospital Copa D'or and Instituto Nacional de Infectologia Evandro Chagas). Blood samples were obtained within the first 72 h from the sepsis diagnosis (sepsis phase) and on the day before ICU discharge (recovery phase). The Control group consisted of age-matched healthy volunteers. We excluded subjects with AIDS, advanced cancer, hematological diseases, and pregnancy. Demographic, clinical, and laboratory data were recorded by local investigators and used for calculating the Simplified Acute Physiological Score (SAPS) -3 and the Sequential Organ Failure Assessment (SOFA) score. Patients were managed by the critical care teams based on the Surviving Sepsis Campaign's recommendations and followed until death or hospital discharge. The study protocol was approved by the National Institute of Infectious Diseases Evandro Chagas Review Board (INI-FIOCRUZ # 059/2011), and the experiments were performed in compliance with this protocol. Written informed consent was obtained from all subjects or their legal surrogates before any study-related procedures.

### Monocyte isolation

Blood samples (20 mL) were drawn into cell preparation tubes with sodium heparin (Vacutainer CPT, BD Biosciences). The tubes were centrifuged at 1700×*g* for 25 min at 20 °C. The mononuclear cell layer (PBMC) was transferred to a conical centrifuge tube and washed twice with PBS (300×*g* centrifugation for 15 min at 20 °C). Monocytes were then separated by an immunomagnetic technique based on anti-CD14 antibodies (EasySep human CD14 positive selection kit, StemCell Technologies): a suspension was prepared at a concentration of 1 × 10^8^ cells/mL in PBS containing 1 mM EDTA and 0.5% (w/v) bovine serum albumin, followed by incubation with tetrameric anti-CD14/anti-dextran antibody complexes (100 μL/mL of cell suspension) for 15 min and with dextran-coated magnetic beads (100 μL/mL of cell suspension) for 10 min at room temperature. Finally, the suspension volume was adjusted to 2.5 mL, and the monocytes (CD14+) were separated by exposure to a magnetic field for 10 min, followed by two washes. The samples were snap-frozen immediately after the cell separation procedure and kept at − 80 °C until further processing for proteomics. Acceptable cell viability (≥ 95%) and purity (≥ 90% CD14+) were assessed by Trypan blue labeling and flow cytometry, respectively (Supplementary Table S3).

### Shotgun proteomics

#### Sample preparation

Isolated monocyte samples were thawed at 4 °C, suspended in 115 μL of Rapigest SF (Waters) at 0.1% (w/v) in 50 mM ammonium bicarbonate, and centrifuged at 20,000×*g* for 30 min at 4 °C. Supernatants were separated, and protein concentration was estimated by absorbance reading at 280 nm (NanoDrop 2000, Thermo Scientific). For each sample, 50 μg of protein were reduced in dithiothreitol (3 h at 37 °C, 10 mM final concentration) and alkylated in iodoacetamide (30 min in the dark at room temperature, 25 mM final concentration). Next, samples were incubated with trypsin (Promega) 1:50 (m/m) at 37 °C for 19 h and at 56 °C for 45 min in a thermoblock (Eppendorf). The reaction was stopped by adding trifluoroacetic acid to a final concentration of 1% (v/v). The tryptic peptides were purified in reversed-phase homemade microcolumns with POROS R2 resin (Applied Biosystems). The peptide concentration was estimated by absorbance reading at 280 nm (NanoDrop 2000, Thermo Scientific), and samples were stored at − 20 °C for mass spectrometry analysis.

#### Mass spectrometry

Samples were subjected to nanoLC-nanoESI MS/MS analysis in an Ultimate 3000 (Dionex) chromatographic system coupled to the Q Exactive Plus mass spectrometer (Thermo). About 1 μg of peptides was initially applied to a 2 cm guard column, followed by fractionation on a 40 cm PicoFritTM Self-Pack column (New Objective) packed with 1.9 μm silica, ReproSil-Pur 120 Å C18-AQ (Dr. Maisch, Germany). Samples were loaded in 0.1% (v/v) formic acid in water (mobile phase A) on the trap column at 2 μL/min, while chromatographic separation occurred at 200 nL/min. Mobile phase B consisted of 0.1% (v/v) formic acid in acetonitrile. Peptides were eluted with a gradient of 2 to 45% B over 32 min, followed by up to 80% B in 4 min. Lens voltage was set to 60 V. Full scan MS mode was acquired with a resolution of 70,000 (FWHM for *m*/*z* 200 and AGC set to 1 × 10^6^). Up to 12 most abundant precursor ions from each MS scan (*m*/*z* 300 to 1500) were sequentially subjected to fragmentation by HCD. Fragment ions were analyzed (MS2 scan) at a resolution of 17,500 and AGC set to 5 × 10^4^. Samples were analyzed in technical triplicate and data acquired using Xcalibur software (version 3.0.63). The mass spectrometry data have been deposited in the ProteomeXchange Consortium via the PRIDE partner repository^35^ under the identifier PXD023938.

### Peptide identification and protein inference

The raw data files were processed and quantified using PatternLab for Proteomics software^36^. Peptide-sequence matching (PSM) was performed using the Comet algorithm^37^ against the protein-centric human database NeXtProt^38^ (downloaded January 29, 2017). A target-decoy strategy was employed. The search parameters were: tryptic and semi-tryptic peptides, with masses between 500 and 5000 Da, up to 2 lost cleavage sites, modifications: carbamidomethylation (Cys), oxidation (Met), and initial tolerance of 40 ppm for precursor ions. PSMs were filtered using the Search Engine Processor (SEPro) module and identifications were grouped by the number of enzymatically cleaved ends, resulting in two distinct subgroups. For each result, each metric's scores (XCorr, DeltaCN, and ZScore) were used to generate a Bayesian discriminator, accepting up to 1% false discovery rate (FDR), estimated by the number of decoy sequence IDs. Results were further filtered to accept only PSMs with a mass error smaller than 5 ppm and protein identifications supported by two or more independent identifications. Proteins identified by a single spectrum (1 hit wonder) with XCorr below 2 were excluded. The final list of mapped proteins was grouped according to maximum parsimony.

### Differentially regulated proteins and in silico functional analysis

Patternlab's TFold module determined differentially abundant proteins based on spectrum counting normalized by NSAF^39^, with a q-value cutoff of 0.05. The area-proportional Venn diagram module displayed all proteins mapped for each condition. For the differential analysis, we included proteins inferred on two or more samples in a group. There was no imputation of missing values. The Molecular Degree of Perturbation (MDP) score of the individual samples was calculated using the MDP tool (http://bioconductor.org/packages/mdp/) from the average of the 25% highest protein z-scores of Sepsis and Recovery groups, calculated using the Control group as reference^40^. The sample MDP from Sepsis and Recovery groups were compared using the Wilcoxon signed-rank test, with *p* < 0.05 defining significance. Heat maps were created using Morpheus (https://software.broadinstitute.org/morpheus). Abundance values were z-score normalized. Hierarchical clustering was based on Euclidean distance. Functional analysis was performed with the InnateDB database pathway analysis tool (http://www.innatedb.com)^18^, using the over-representation analysis (Hypergeometric algorithm, Benjamini- Hochberg correction with *p* < 0.05 defining significance) method with Reactome pathway annotations^19^. The enrichment network from Reactome pathways and chord graph were created using NetworkAnalyst 3.0^41^.

## Supplementary Information

Supplementary Information 1.

Supplementary Information 2.

Supplementary Information 3.
