# Supplementary material for: Proteomics reveals disturbances in the immune response and energy metabolism of monocytes from patients with septic shock
Source: Sci Rep. 2021 Jul 26;11:15149. doi: 10.1038/s41598-021-94474-0 (PMC8313678; doi:10.1038/s41598-021-94474-0)
Supplement: Supplementary file 1 — Supplementary Information 1. [file 41598_2021_94474_MOESM1_ESM.docx]

**Proteomics reveals disturbances in the immune response and energy metabolism of monocytes from patients with septic shock**

**Pedro Mendes de Azambuja Rodrigues, Richard Hemmi Valente, Giselle Villa Flor Brunoro, Helder Takashi Imoto Nakaya, Mariana Araújo-Pereira, Patricia Torres Bozza, Fernando Augusto Bozza, Monique Ramos de Oliveira Trugilho**

Supplementary Table S1. Demographic characteristics, disease severity, and outcomes of septic patients and controls

|  | **Sepsis / Recovery**  **(N=9)** | **Control**  **(N=6)** |
| --- | --- | --- |
| **Age** | **67.7 (**± **12.3)** | **67.8 (**± **4.9)** |
| **Sex (male/female)** | **4/5** | **3/3** |
| **Infection site:** |  |  |
| **- Lungs** | **4** | **-** |
| **- Urinary tract** | **3** | **-** |
| **- Abdominal** | **1** | **-** |
| **- Skin** | **1** |  |
| **Microbiology:** |  |  |
| **- Gram-negative** | **5** | **-** |
| **- Gram-positive** | **1** | **-** |
| **- Polymicrobial** | **1** | **-** |
| **- Not detected** | **2** | **-** |
| **SAPS 3** | **58.6 (**± **12.9)** | **-** |
| **SOFA (Sepsis)** | **11.3 (**± **3.1)** | **-** |
| **SOFA (Recovery)** | **1.4 (**± **1.3)** |  |
| **Mechanical ventilation** | **6** | **-** |
| **Vasopressor** | **9** | **-** |
| **Renal replacement therapy** | **3** | **-** |
| **Length of ICU stay** | **19.8 (**± **9.1)** | **-** |
| **Deaths in 28 days** | **0** |  |
| **Hospital deaths** | **1** | **-** |

Variables expressed as mean ± standard deviation. SOFA: Sequential Organ Failure Assessment; SAPS: Simplified Acute Physiologic Score

Supplementary Table S2. Absolute number of identified peptides and inferred proteins in the experimental groups

|  | **SEPro Fusion (Combined FDR)** | | |
| --- | --- | --- | --- |
|  | **Control** | **Sepsis** | **Recovery** |
| ***Peptide-spectrum Match* (PSM)** | **283,319 (0.4%)** | **368,971 (0.05%)** | **413,482 (0.05%)** |
| **All peptides** | **40,447 (0.08%)** | **43,016 (0.1%)** | **48,339 (0.11%)** |
| **Unique (proteotypic) peptides** | **21,899** | **23,830** | **26,498** |
| **All proteins** | **6,652 (0.12%)** | **6,618 (0.12%)** | **7,043 (0.17%)** |
| **Maximum parsimony proteins** | **3,454** | **3,496** | **3,716** |
| **Proteins with at least one unique (proteotypic) peptide** | **2,908** | **2,973** | **3,191** |

*Statistical filtering and compilation using the SEPro module of PatternLab for Proteomics*

Supplementary Table S3. Parameters derived from sample preparation

| **Monocytes** | **Control** | **Sepsis** | **Recovery** |
| --- | --- | --- | --- |
| **Number (x10^6^)** | **2.43 (± 1.87)** | **2.11 (± 0.27)** | **3.62 (± 0.54)** |
| **Viability (%)** | **96.3 (± 1.22)** | **96.66 (± 1.66)** | **95.32 (± 0.97)** |
| **Purity (%)** | **94 (± 1.28)** | **95.15 (± 1.48)** | **94.62 (± 1.03)** |
| **Proteins (mg/ml)** | **1.41 (± 0.58)** | **1.76 (± 0.62)** | **1.95 (± 0.52)** |
| **Proteins (µg)** | **70.5 (± 29.01)** | **87.99 (± 31.21)** | **97.56 (± 26.47)** |


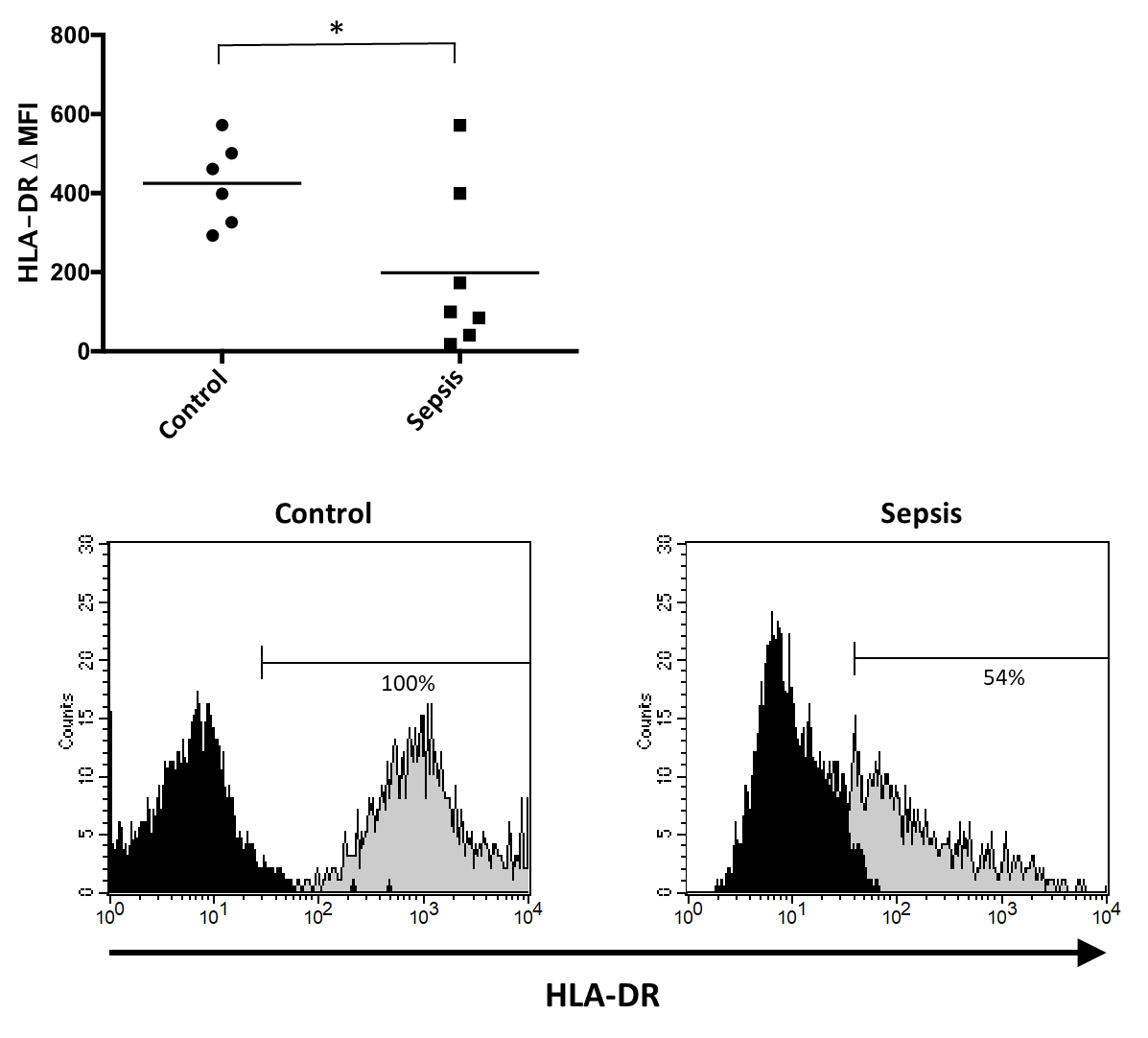


**Supplementary Figure S1. HLA-DR expression in sepsis patients and healthy controls**. Monocytes (CD14+, PerCP-Cy5.5) were analyzed by flow cytometry for the expression of HLA-DR (PE) on the cell surface. Above, the horizontal lines represent the averages. Below, representative histograms of the experiments are shown, with the percentage of positive cells (grey) in relation to the indicated isotype control (black). Δ MFI: median fluorescence intensity (MFI) of the test subtracted from the MFI of the isotypic control. * p < 0.05 (Student's T test).

**Supplementary Materials and Methods**

**Measurement of monocyte HLA-DR expression by flow cytometry**

Blood samples (100 μL) collected EDTA tubes (Vacutainer, BD biosciences) were labeled with anti-CD14 PeCP-Cy5.5 (5 μL - 0.5 μg, eBioscience) combined with anti- HLA-DR PE (5 μl - 0.25 μg, eBioscience) or isotype control for 15 minutes at room temperature. Then, they were incubated with 2 ml of a red cell lysis buffer (BD FACS Lysing Solution, BD Biosciences) for 15 minutes at room temperature, washed three times with PBS, fixed in 1% paraformaldehyde (m/v) and analyzed in less than 2 hours. Event acquisition and analysis was performed in a three-color cytometer (BD FACSCalibur, BD Biosciences), using the CellQuest Pro software (BD Biosciences). Monocyte gating was performed according to the characteristics of side scatter combined with anti-CD14 antibody labeling.
